# Factors related to self-rated health: a survey among patients and their general practitioners

**DOI:** 10.1080/02813432.2021.2022341

**Published:** 2022-05-19

**Authors:** Mona Kjeldsberg, H. Tschudi-Madsen, D. Bruusgaard, B. Natvig

**Affiliations:** Faculty of Medicine, Institute of Health and Society, University of Oslo, Oslo, Norway

**Keywords:** Self-rated health, symptom reporting, diagnoses, life stressors, general practice

## Abstract

**Objective:**

To explore associations between general practice patients’ SRH and symptoms, diagnoses, chronic conditions, unexplained conditions, and life stressors.

**Design:**

A cross-sectional study. Data were collected from GP and patient questionnaires.

**Setting:**

General practices in Southeast Norway.

**Subjects:**

47 general practitioners (GPs) who included 866 consecutive patients.

**Main outcome measures:**

SRH was measured with a single question from the COOP-WONCA overall health chart and dichotomized into good/poor SRH. Binary logistic regression models were used in the analyses.

**Results:**

Poor SRH was reported by 48% of the patients in the past week. A higher prevalence of poor SRH was found for women, middle-aged, recipients of social security grants, patients diagnosed with asthenia, lower back pain, and depression/anxiety, and for patients with reported life stressors and unexplained conditions. We found an almost linear association between the number of symptoms and the likelihood of reporting poor SRH. The probability of reporting poor SRH increased along with an increasing number of symptoms for common diagnoses. In a multivariate analysis, the only number of symptoms, being in receipt of social security grants and being retired was associated with poor SRH.

**Conclusion:**

The likelihood of reporting poor SRH increased with an increasing number of symptoms, partly independent of the diagnosis given by GPs. This result coincides with our previous findings of a strong association between the number of symptoms, function, and health. The symptom burden thus appears to be an important factor for SRH among patients in general practice.

**KEY POINTS**
- There is a high prevalence of poor SRH in general practice patients.
- The likelihood of reporting poor SRH is partly independent of the diagnosis given.
- The number of symptoms was the factor strongest associated with poor SRH.

## Introduction

SRH is considered to be an important predictor for general well-being, in addition, to predict morbidity and mortality, and is therefore suggested to be implemented in clinical practice in primary care [1]. As established by the World Health Organization, health is a much broader concept than the mere absence of disease [2], influenced not only by medical but also social, psychological, and lifestyle factors [3]. Self-rated health (SRH) should thus reflect an individual’s health status in this broad sense.

SRH is associated with several sociodemographic factors like gender [4], age [5], employment status, and educational level [4]. In addition, physical function [6], medically unexplained conditions [7], life stressors, and negative life events [8] are associated with SRH.

Diagnoses play a central role in general practitioners’ (GPs’) assessment of patients’ function and workability [9]. Some disorders, such as depression and lower back pain, seemingly affect SRH more than others [10]. Yet, patients’ SRH is not necessarily reflected in the given diagnostic labels. A study on SRH among adolescents in Norway showed that being diagnosed with a medical condition—or having specific mental or somatic health symptoms—was of less importance for SRH four years later than their functional status and subjective, the general sense of well-being [11].

Poor SRH l is associated with having chronic diseases, such as rheumatoid arthritis, cancer, and neurological disease [12]. However, symptoms, like tiredness and pain, may contribute more to the total burden of poor SRH at a population level than chronic diseases. Others have found that poor SRH in primary care patients largely is attributable to symptoms like pain and lack of energy [13].

Our research group has published several studies on symptom reporting and multi symptomatology. Results from the Ullensaker population study showed that a high number of pain symptoms was associated with poor health and low function [14], and constituted a risk for future work disability [15].

Previous studies of associations between SRH, symptoms, and diagnoses have tended to look at preselected diagnoses or patient-reported diagnoses [12]. In this article, we wanted to link responses from questionnaires to patients and their GPs. The aim was to describe associations between SRH and the number of symptoms, as well as diagnoses and several other variables in GP patients.

## Method

We have used data from a cross-sectional study among GPs and their patients, with the main focus on symptoms and multi symptomatology. The study was conducted in Oslo and Akershus counties, Norway. The inclusion period was from June 2010 to January 2012. The GPs were recruited from counselling group meetings in general practice. After a short introduction, 66 GPs were invited, and among them, 47 GPs accepted the invitation to participate in the study. Each GP was asked to include 20 consecutive patients aged 18 years or more on a random day in practice, regardless of the reason for the encounter. If a GP included <20 adult patients on a practice day, the inclusion should continue the next practice day. Information regarding patients declining to participate was not collected. Corresponding questionnaires for the GPs and their patients were to be completed directly after the consultation. The answers were linked by serial numbers. Linked questionnaires from 882 pairs of patients and GPs were returned, out of which 866 pairs had complete data on all variables and were included in the study.

### Dependent variable

The dependent variable ‘self-rated-health’ (SRH) was measured by the COOP-WONCA overall health chart on a five-point scale [16]. The question on the chart was; ‘How would you evaluate your own physical and mental health during the past seven days?’ with the response categories being ‘very good’, ‘good’, ‘average’, ‘poor’, and ‘very poor’. We dichotomized the variables into ‘good’ (very good + good) and ‘poor’ (average + poor + very poor) with poor SRH as the dependent variable.

### Independent variables

Each patient filled in a checklist of 38 symptoms experienced during the past seven days. The symptom checklist consists of 28 symptoms from the Subjective Health Complaint Inventory (SHC) [17] and 10 symptoms from the Standardised Nordic Questionnaire (SNQ) [18]. The question asked was: ‘Have you experienced any of these symptoms during the past seven days?’ We counted symptoms reported and created five groups of symptoms of approximately equal size in our data: 0–2, 3–4, 5–7, 8–11, and 12+. To record whether the patients suffered from or had considered that they might be suffering from selected medically unexplained conditions, the following question was posed: ‘Do you suffer from, or have you considered whether you suffer from, one or more of the following conditions: amalgam poisoning, candida syndrome, electromagnetic hypersensitivity syndrome, fibromyalgia, chronic fatigue syndrome/myalgic encephalopathy, food intolerance, burnout syndrome or irritable bowel syndrome?’ The conditions were not further defined or explained, and we did not define a time window but included any consideration they might have, present or past.

We addressed life stressors with the following question: ‘Do you experience that any of the following issues have had a negative influence on your present health?: (i) work situation, (ii) experiences in childhood/adolescence, (iii) family issues, (iv) economic issues, and (v) other serious life events’.

Employment status was registered in the following eight categories; employed, homeworker, student, unemployed, short-term sick leave, long-term sick leave, disability pension and retired. We collapsed employed, homeworker, and student into ‘employed’, short- and long-term sick leave, disability pension, and unemployed into ‘social security grants’, while ‘retired’ was kept as a separate category.

The GPs registered the main diagnosis in the consultation by using codes from the International Classification of Primary Care (ICPC)-2 system [19] or as text. The texted diagnoses were labelled with ICPC codes before the analyses; 321 different ICPC-2 diagnoses are recorded. We selected the most frequent single diagnoses for further analysis: hypertension (K85, K86, *n* = 56), depression/anxiety (P01, P03, P74, and P76, *n* = 37), asthenia (A04, *n* = 34), diabetes (T89, T90, *n* = 27) and lower back pain (L02, L03, L84, and L85, *n* = 24).

In addition to the main diagnosis, the GPs registered prevalent chronic conditions, and we created a sum score of the number of chronic conditions (0–3+).

### Statistical methods

Frequencies and percentages are used to describe the prevalence of symptoms, and Chi-squared tests were used to compare the groups. *p-*Value <0.05 was accepted as statistically significant. Dichotomized variables relating to SRH were analysed using binary logistic regression, and probability curves were obtained from this model. We performed two different regression models with poor SRH as an outcome variable. The possibility for multi-collinearity was checked before the multivariate analyses were performed. We did not have collinearity problems in our analyses (all the independent variables had VIF <10). Regression model I was performed with SRH as the dependent variable with all the variables included, where the symptoms were treated as a sum variable classified into five groups. In a separate model (II), SRH was the dependent variable and the individual symptoms were the independent variables, controlling for age, gender, and the number of symptoms. Results of multivariate analyses are reported as the odds ratio (OR) and 95% confidence interval (CI). IBM SPSS statistics (v. 25; IBM Corp., Armonk, NY, USA) was used for all analyses.

## Results

In total, 1024 questionnaires were handed out. Matched responses from the GPs and the patients were included in the final analysis (*n* = 866), giving an overall, matched response rate of 84.6%.

Poor SRH the last week was reported by 47.8%, a summary of the categories average (24.7%), poor (19.9%), and very poor SRH (3.2%) (Table 1).

**Table 1.** Self-rated health (SRH) in the last 7 days by gender.

	Total (*n* = 866)		Men (*n* = 307)		Women (*n* = 559)	
Self-rated health	*n*	%	*n*	%	*n*	%
Very good	141	16.3	55	17.9	86	15.4
Good	311	35.9	116	37.8	195	34.9
Average	214	24.7	76	24.8	138	24.7
Poor	172	19.9	48	15.6	124	22.2
Very poor	28	3.2	12	3.9	16	2.9

The independent variables are presented in Table 2, and the individual symptoms in Table 3. In the bivariate analysis, more women than men reported poor SRH, although this was not significant (49.7 *vs.* 44.3%). Middle-aged respondents reported more often poor SRH than the younger and older age groups (Table 2).

**Table 2.** Characteristics of the patients in the study, and their SRH in the past 7 days.

	*n* = 866	Poor health	
	%	%	*p*-Values
Total		47.8	
Gender			0.126
Men	35.5	44.3	
Women	64.5	49.7	
Age, years			0.029
18–39	36.7	48.7	
40–59	34.9	52.3	
60+	28.4	41.1	
Civil status			0.007
Married	66.4	44.5	
Not married	33.6	54.3	
Education level			0.013
10–13 years	54.5	52.1	
University 1–4 years	27.7	44.6	
University > 4 years	17.8	39.6	
Employment status			<0.001
Employed	52.7	34.4	
Social security grants	29.3	76.4	
Retired	18.0	40.4	
Prevalent diagnoses			<0.001
Hypertension	6.5	30.4	
Depression/anxiety	4.3	67.6	
Asthenia	3.9	73.5	
Diabetes	3.1	44.4	
Lower back pain	2.7	69.6	
Other	79.6	46.3	
Chronic conditions			0.001
0	35.3	39.9	
1	35.5	48.2	
2	18.8	55.8	
3+	10.4	58.9	
Number of symptoms			<0.001
0–2	17.9	18.1	
3–4	17.2	20.8	
5–7	24.2	46.2	
8–11	19.4	61.9	
12+	21.2	83.7	
Unexplained conditions*			
Burnout syndrome	17.4	72.2	<0.001
Irritable bowel syndrome	16.2	65.0	<0.001
Food intolerance	11.3	66.3	<0.001
Fibromyalgia	9.1	82.3	<0.001
CFS/ME	7.6	80.3	<0.001
Candida syndrome	3.0	65.4	0.068
Amalgam poisoning	3.2	53.6	0.535
Electromagnetic hypersensitivity	1.5	76.9	0.034
Life stressors*			
Work situation	27.9	59.9	<0.001
Family	17.6	67.1	<0.001
Economy	12.7	69.1	<0.001
Adverse childhood experiences	11.5	75.0	<0.001
Serious life events	10.2	69.3	<0.001

CFS: chronic fatigue syndrome; ME: myalgic encephalopathy.

The *p*-values were calculated using Pearson’s Chi-squared test.

*These categories are not mutually exclusive.

**Table 3.** The association between independent variables and poor SRH in the past 7 days.

		Adjusted	
	OR	95% CI	*p*-Value
Gender (ref. men)			
Women	0.91	(0.64–1.30)	0.608
Age, years (18–29)			
40–59	0.76	(0.51–1.15)	0.191
60+	0.38	(0.20–0.72)	0.003
Civil status (married)			
Not married	1.12	(0.79–1.59)	0.515
Education level (10–13 years)			
University 1–4 years	0.94	(0.63–1.39)	0.752
Universit*y* > 4 years	0.82	(0.52–1.30)	0.392
Employment status (employed)			
Social security grants	4.21	(2.78–6.38)	0.000
Retired	2.25	(1.14–4.45)	0.020
Prevalent diagnoses (other)			
Hypertension	0.62	(0.30–1.26)	0.349
Depression/anxiety	0.83	(0.36–1.90)	0.544
Asthenia	1.55	(0.62–3.84)	0.656
Diabetes	1.33	(0.53–3.38)	0.183
Lower back pain	2.08	(0.74–5.84)	0.165
Chron. conditions (0)			
1	1.17	(0.79–1.74)	0.430
2	1.56	(0.93–2.62)	0.095
3+	1.13	(0.60–2.12)	0.712
Number of symptoms (0–2)			
3–4	1.10	(0.61–2.0)	0.757
5–7	3.30	(1.96–5.55)	0.000
8–11	5.79	(3.34–10.04)	0.000
12+	16.48	(8.92–30.46)	0.000
Unexplained conditions (none)			
1+	1.32	(0.93–1.88)	0.117
Life stressors (none)			
1+	1.14	(0.81–1.61)	0.458

*Note*. Binary logistic regression analysis was performed.

Low education was associated with poor SRH. Patients who received social security grants had a high prevalence of poor SRH (76.4%), while only 34.4% of the employed persons reported poor SRH. Poor SRH was reported by patients with diagnoses of asthenia (73.5%), lower back pain (69.6%), and depression/anxiety (67.6%), while only 30.4% of those with hypertension reported poor SRH. Patients with at least one chronic condition had worse SRH than those with no such condition. All the life stressors and all the unexplained conditions except amalgam poisoning and candida syndrome were significantly associated with poor SRH.

Of the 38 different symptoms, all the individual symptoms except urinary problems and leg cramps were significantly associated with poor SRH (*p* < 0.05; data not shown).

**Figure 1.**
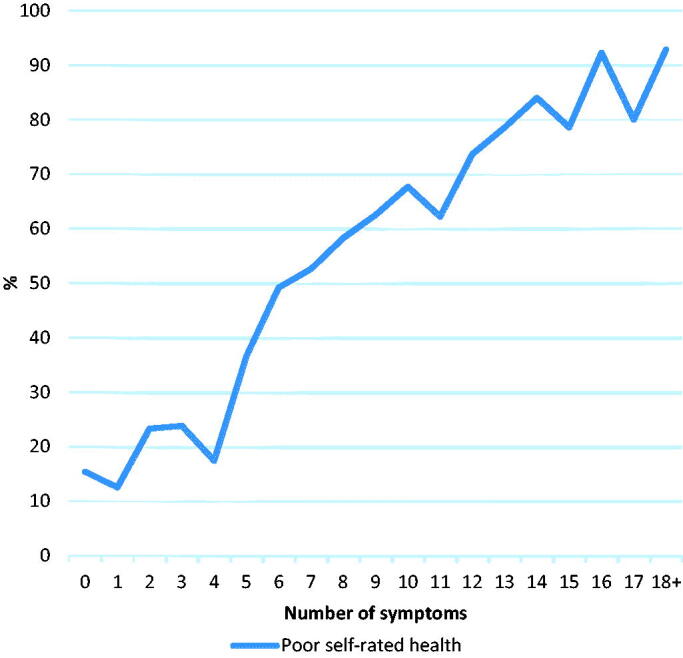
Percentage of patients reporting poor SRH according to the number of symptoms in the past week.

There was an almost linear relationship between the prevalence of poor SRH and the number of symptoms, with 83.7% of patients with 12 or more symptoms reporting poor SRH, while only 18.1% of patients with two or fewer symptoms reported poor SRH (Figure 1).

In a multivariate model (regression model I), when controlling for all the independent variables, the number of symptoms was the factor strongest associated with poor SRH (OR 12+ symptoms 16.5; 95% CI 8.9–30.5, compared to 0–2 symptoms). In addition, being in receipt of social security grants (OR 4.2; 95% CI 2.8–6.4) and being retired (OR 2.3; 95% CI 1.1–4.5) were associated with poor SRH. The age group 60+ reported better SRH (OR 0.4; 95% CI 0.2–0.7) than the other age groups (Table 3). In this model, the most prevalent diagnoses, the number of chronic conditions, reporting at least one life stressor, reporting at least one unexplained condition, civil status, and education level no longer gave significant contributions to poor SRH.

**Figure 2.**
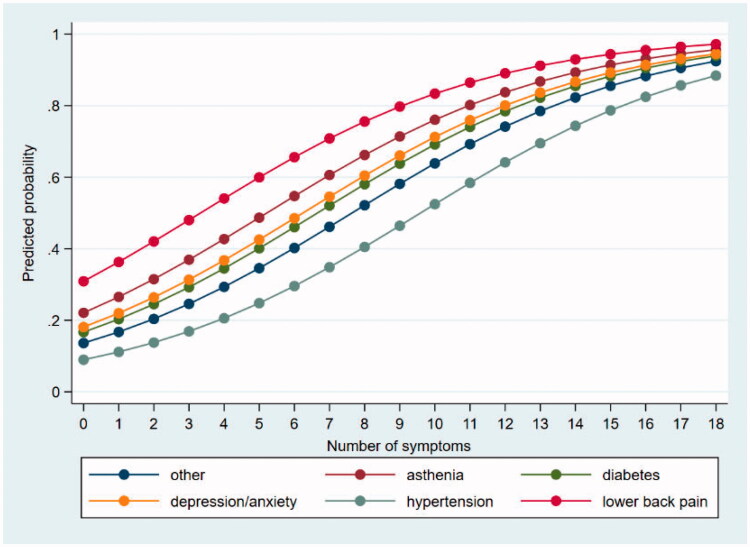
Predicted probability of poor self-rated health according to the number of symptoms reported by the patients and the most prevalent diagnoses given by their GPs.

A separate probability analysis based on results from the regression model I showed that the predicted probability of reporting poor SRH increased with an increasing number of symptoms for all the selected diagnoses (Figure 2).

In regression model II, where individual symptoms were used as the independent variable, controlling for gender, age, and the number of symptoms, the following symptoms remained significantly associated with poor SRH: tiredness (OR 2.5; 95% CI 1.7–3.7), hip pain (OR 2.4; 95% CI 1.5–3.7), depression (OR 2.2; 95% CI 1.3–3.7), problems concentrating (OR 2.1; 95% CI 1.2–3.4), anxiety (OR 1.7; 95% CI 1.0–2.9), headache (OR 1.6; 95% CI 1.1–2.3) and lower back pain (OR 1.5; 95% CI 1.1–2.2; Table 4).

**Table 4.** Individual symptoms reported in the past week and associations with poor SRH.

Symptoms	*n*	Adj. OR	95% CI	*p*-Value
Tiredness	270	2.4	(1.7–3.4)	**<0.001**
Lower back pain	231	1.3	(1.1–2.2)	**0.043**
Headache	216	1.4	(1.2–2.5)	**0.025**
Neck pain	212	0.7	(0.5–1.0)	0.067
Sleep problems	200	1.2	(0.8–1.7)	0.329
Shoulder pain	190	0.8	(0.6–1.2)	0.298
Problems concentrating	157	1.9	(1.2–3.0)	**0.004**
Hand/wrist pain	146	0.9	(0.6–1.4)	0.694
Dizziness	133	1.0	(0.6–1.5)	0.881
Depression	132	2.2	(1.4–3.4)	**0.001**
Infection	131	1.1	(0.8–1.5)	0.683
Hip pain	130	2.3	(1.5–3.6)	**<0.001**
Cold hands/feet	130	0.8	(0.5–1.2)	0.263
Knee pain	128	0.8	(0.5–1.1)	0.206
Anxiety	125	1.8	(1.2–2.9)	**0.007**
Upper back pain	124	0.7	(0.5–1.1)	0.107
Ankle/foot pain	123	0.9	(0.6–1.3)	0.457
Flatulence/bowel gas	120	0.8	(0.5–1.2)	0.334
Memory problems	108	1.2	(0.7–1.9)	0.531
Hot flushes	106	1.1	(0.7–1.7)	0.650
Breathing difficulties	99	1.4	(0.9–2.2)	0.190
Diarrhoea	97	1.1	(0.7–1.6)	0.832
Heart burn/dyspepsia	93	0.8	(0.5–1.3)	0.431
Dry eyes	91	1.0	(0.6–1.5)	0.875
Eczema	85	0.7	(0.4–1.2)	0.314
Palpitations	80	0.8	(0.5–1.4)	0.449
Tinnitus	68	0.6	(0.4–1.0)	0.069
Chest pain	65	1.0	(0.6–1.8)	0.990
Elbow pain	64	1.0	(0.6–1.6)	0.909
Oedema	62	1.4	(0.7–2.7)	0.303
Vomiting	60	0.9	(0.5–1.6)	0.746
Leg cramps	60	0.6	(0.3–1.4)	0.280
Fasciculation/twitches	48	0.6	(0.3–1.1)	0.072
Allergy	48	0.7	(0.4–1.3)	0.238
Constipation	44	0.5	(0.3–1.4)	0.302
Urinary problems	37	0.6	(0.3–1.1)	0.119
Sight problems	31	0.6	(0.3–1.3)	0.183
Fainting	13	1.3	(0.3–6.0)	0.718

Estimates of ORs with their 95% CIs obtained from binary logistic regression model II adjusted for age, gender and the number of symptoms. Significant differences (*p* < 0.05) are marked in bold.

## Discussion

### Summary of the main findings

Poor SRH was reported by 48% of the patients. The factor most strongly associated with poor SRH was the number of symptoms, followed by being the recipient of social security grants, and being retired. Seven of the 38 individual symptoms were also associated with poor SRH. In the multivariate analysis, the prevalent diagnoses, the numbers of chronic conditions, life stressors, and unexplained conditions were not associated with poor SRH, while the oldest age group still reported better SRH than the other age groups.

### Strengths and weaknesses of the study

A strength of our study is that it was based on consecutive, unselected, adult patients. Any selection bias of patients through their GPs should be minimal because Norwegian GPs do not select patients on their lists. We found a high number of different ICPC-2 codes (*n* = 321). Almost the same extent of different diagnoses was found in a recent Norwegian study [20]. This indicates that we had a relatively representative study sample. To measure self-rated health, we used the COOP-WONCA overall health chart, which has been validated and has good reliability [16]. The answers to various SRH instruments are highly correlated [6], and they are validated to be strong predictors of morbidity, mortality, and disability retirement [21].

As our objectives were largely explorative and we wanted to assess associations between poor SRH and a diversity of other variables, we did not perform power calculations of the study size. Our pooling of three response categories, average + poor + very poor, into poor SRH can be questioned. We chose this cut-off due to our preconception that the patients’ perception of the wording of the middle category, ‘average’, probably is closer to poor than good SRH [4]. Although not validated, this dichotomization is used in several other studies [4]. We have modified the COOP-WONCA time window from the original two weeks to one week, to match the symptom checklist window from the SHC and SNQ. However, we think it is unlikely that our modification would have affected the results significantly, as symptom reporting, in general, seems to be rather stable over time [22].

Although we selected the most prevalent diagnoses for the analyses, each diagnosis was relatively uncommon. We did not ask for details of the severity of symptoms, but including also minor complaints has been found to be of importance when assessing symptom load and health outcome [14].

### Findings in relation to other studies

We found a relatively high prevalence of poor SRH in this general practice population. Different labelling of the SRH categories may affect how patients respond when rating their own health. This is illustrated by the middle category in the different five response versions of SRH is labelled ‘good’ [5], fair [4], ‘average’ [23], or ‘alright’ [21]. The cut-offs for dichotomization of the five-item version of SRH also vary and will have a large impact on the prevalence rates of poor SRH. The middle SRH category, which in our study made up nearly 25% of the responses [5], is in some studies pooled with good health [7] and in others with poor health [4], or it may also be retained as a separate category [5]. A different pooling in our study, with poor + very poor pooled into poor SRH, would have given a prevalence rate of 23% poor SRH. A recent Norwegian study in general practice found that 35% of the patients reported excellent or very good SRH, 39% good and 26% reported fair or poor SRH [5]. Population-based studies have found a somewhat lower prevalence of poor SRH [4] than we found in general practice patients, which is to be expected. The finding of a small, though statistically insignificant, the gender difference in SRH in our study was also consistent with previous studies [24].

The oldest age group reported better health than the rest of the population, which is a result that seems counterintuitive. Several studies have found that higher age is associated with poor SRH [4,5,25]. Some of this association between age and poor SRH can be related to an increasing prevalence of chronic diseases with age. However, the results from other studies are inconsistent. In one study age was found to affect SRH more than the presence of chronic conditions [26]. Other studies have found that age in itself was not associated with poor SRH [23]. A recent Norwegian study even found that increasing age was associated with better health, in line with our results [27]. In a Finnish study, the individuals reported better health at a 10-year follow-up, despite also reporting more diseases [28]. Probably elderly patients and patients with chronic illness learn to cope, or change their expectations over time, and might then perceive their health as improved, even though others would see their health situation as unaltered [29]. The divergent results can to some extent be attributed to methodological differences.

The strong association we found between poor SRH and receiving social security benefits was retained even after controlling for the number of symptoms. This association is well-known [15,23].

Previous studies have found that the number of symptoms, together with symptom severity, are closely correlated with health outcomes among patients in primary and secondary care [30]. Assessment of symptom counts alone, rather than also looking at the severity of symptoms, has been seen as a possible limitation in symptom research. However, the almost linear relationship we found between the number of symptoms and SRH is supported by other studies. A large population study in the UK found the total symptom score to be the strongest correlate of SRH [23]. Our research group has also previously found SRH to be strongly associated with the number of symptoms even when adjusting for the type or severity of the symptoms [14].

The association between the number of chronic conditions and SRH in our study was surprisingly weak. In part, this might be because we used a simple count of chronic conditions rather than a measure that also could capture the severity of disorders. Individuals with chronic conditions are often described to have a higher risk of reporting poor SRH and to experience more limitations and functional impairment compared with individuals with no chronic condition [31]. On the other hand, Kroenke et al. found a weak association between the number of disorders and health outcomes [30]. This is consistent with results from another study where individuals with limitations in daily activities reported poorer health than those without such limitations, regardless of the number of chronic conditions [32].

We found that patients with asthenia, depression/anxiety, and lower back pain diagnoses reported reduced SRH, compared with the whole patient group. Patients with these diagnoses are known to report many symptoms [33], while patients with, for example, well-controlled diabetes or hypertension have few symptoms with less impact on SRH. Overall, this indicates that it is the consequences of disease rather than the disease itself that affect how people rate their health [34]. This also fits with SRH being a better predictor of disability retirement than a given diagnosed disease [35].

In our study, patients reporting at least one unexplained condition reported significantly reduced SRH. This finding might be explained by that patient with such diagnoses report a high number of symptoms [36], as this result was no longer significant when controlling for the other variables. Further, we found an association between negative life events and poor SRH, as in several other studies [23,37].

Many of the associations between our independent variables and poor SRH disappeared in the multivariate analyses, as a result of the very strong association between poor SRH and the number of symptoms. When adjusting for the number of symptoms, only a few of the individual symptoms remained significantly associated with poor SRH. This might be because reporting one symptom increases the probability of reporting several other symptoms [36]. Our findings that common symptoms, such as tiredness, lower back pain, and headache are associated with poor SRH, are in accordance with recent studies [5]. Especially for tiredness and pain symptoms [12,13], studies have indicated a strong correlation with SRH [23].

It is previously demonstrated that the prognosis for poor health in a patient increases with an increasing number of symptoms [15,38] and that the number of symptoms may be better at predicting function than the diagnoses given [22]. Further, lasting symptoms that cannot be attributed to a definite diagnosis tend to have a large negative impact on health and functioning [39].

With an increasing focus on patient-centred medicine and patient participation, we need additional tools to understand and describe our patients’ problems and needs. We suggest that both SRH and ‘number of symptoms’ are worth more attention, both in clinical work and in future research.

## Conclusion

Self-rated health was strongly associated with the number of symptoms, partly independent of the diagnoses given by the GPs. This supports our previous findings of health and function being closely linked to the number of symptoms in the population. Future approaches to study SRH among patients in general practice might benefit from including a broad spectrum of patient-reported symptoms.
